# Prognostic value of IDH2R140 and IDH2R172 mutations in patients with acute myeloid leukemia: a systematic review and meta-analysis

**DOI:** 10.1186/s12885-023-11034-7

**Published:** 2023-06-09

**Authors:** Yao Qin, Kai Shen, Ting Liu, Hongbing Ma

**Affiliations:** Department of Hematology, West China Hospital, Sichuan University, No. 37 Guo Xue Xiang, Wuhou District, Chengdu, 610041 Sichuan China

**Keywords:** IDH2R140 mutation, IDH2R172 mutation, Acute myeloid leukemia, Prognostic value, Systematic review, Meta-analysis

## Abstract

**Background:**

Whether isocitrate dehydrogenase 2 (IDH2) R140 and R172 gene mutations affect the prognosis of patients with acute myeloid leukemia (AML) is controversial. Here, we performed a meta-analysis to assess their prognostic value.

**Methods:**

Eligible studies were systematically searched from PubMed, Embase, the Cochrane Library and Chinese databases up to June 1, 2022. We extracted the hazard ratios (HRs) and their 95% confidence intervals (CIs) of overall survival (OS) and progression-free survival (PFS) to carry out a meta-analysis by a fixed effect model or random effect model according to the heterogeneity between studies.

**Results:**

A total of 12725 AML patients from 11 studies were included in this meta-analysis, of which 1111 (8.7%) and 305 (2.4%) had IDH2R140 and IDH2R172 mutations, respectively. The results revealed that both IDH2R140 and IDH2R172 mutations had no significant effect on OS (IDH2R140: HR = 0.92, 95% CI: 0.77–1.10, *P* = 0.365; IDH2R172: HR = 0.91, 95% CI: 0.65–1.28, *P* = 0.590) or PFS (IDH2R140: HR = 1.02, 95% CI: 0.75–1.40, *P* = 0.881; IDH2R172: HR = 1.31, 95% CI: 0.78–2.22, *P* = 0.306) in AML patients. Subgroup analysis of AML patients with IDH2R140 mutation revealed that studies from the USA (HR = 0.60, 95% CI: 0.41–0.89, *P* = 0.010) and ≤ 50 years old (HR = 0.63, 95% CI: 0.50–0.80, *P* = 0.000) had longer OS. However, studies from Sweden (HR = 1.94, 95% CI: 1.07–3.53, *P* = 0.030) had shorter OS. Meanwhile, subgroup analysis of AML patients with IDH2R172 mutation showed that studies from Germany/Austria (HR = 0.76, 95% CI: 0.61–0.94, *P* = 0.012) and from Sweden (HR = 0.22, 95% CI: 0.07–0.74, *P* = 0.014) had longer OS, whereas studies from the UK (HR = 1.49, 95% CI: 1.13–1.96, *P* = 0.005) and studies with nonmultivariate analysis of data type (HR = 1.35, 95% CI: 1.06–1.73, *P* = 0.014) had shorter OS. In addition, our study also found that patients with IDH2R140 mutation had significantly longer OS (HR = 0.61, 95% CI: 0.39–0.96, *P* = 0.032) and PFS (HR = 0.31, 95% CI: 0.18–0.52, *P* = 0.021) than patients with IDH2R172 mutation, despite some degree of heterogeneity.

**Conclusions:**

This meta-analysis demonstrates that IDH2R140 mutation improves OS in younger AML patients and that the prognostic value of IDH2R172 mutation is significantly heterogeneous. Differences in region and data type have a significant impact on the prognosis of AML patients with IDH2R140 and/or IDH2R172 mutations. Additionally, AML patients with IDH2R140 mutation have a better prognosis than those with IDH2R172 mutations, albeit with some degree of heterogeneity.

## Background

Acute myeloid leukemia (AML) is a hematologic malignancy with highly significant heterogeneity [1]. According to cytogenetic abnormalities of the European LeukemiaNet 2017, the prognosis of AML can be divided into three risk stratifications: favorable, intermediate, and adverse risk groups [2]. Although it is possible to predict the clinical outcome of most AM patients with favorable or adverse karyotypes [3], patients with intermediate risk karyotypes (IR-AML) require additional molecular markers to determine their prognosis and guide personalized therapy. Fortunately, several novel mutations with prognostic implications have been identified in AML over the past few decades. These include internal tandem duplications (ITDs) in the fms-like tyrosine kinase 3 (FLT3) gene, which result in a poor prognosis, and mutations in the nucleophosmin 1 (NPM1) gene, which have a good prognosis in the absence of FLT3-ITDs [4–7]. Next-generation sequencing (NGS) technology offers new opportunities to discover additional mutational profiles in the AML genome, such as the genes encoding DNA methyltransferase 3A (DNMT3A), isocitrate dehydrogenase 1 and 2 (IDH1/2), and Tet oncogene family member 2 (TET2) [8], which are key to DNA methylation modification and are involved in the pathogenesis of leukemia [9, 10]. In summary, mutation analysis can help improve risk stratification and provide a better basis for treatment decisions.

Mutations in the IDH 1 and 2 genes have been identified in AML [11]. IDH2 mutations are present in approximately 10% of adult AML cases and occur at either residue R140 or R172 [12–14]. The IDH family consists of three isoforms, IDH1, IDH2 and IDH3, of which IDH1 is located in the cytosol, while IDH2 and IDH3 are located in the mitochondria and are normally involved in citrate metabolism in the tricarboxylic acid cycle [15]. Under normal conditions, IDH enzymes catalyze the conversion of ﻿isocitrate to α-ketoglutarate (αKG). The presence of IDH mutant enzymes results in the abnormal production of 2 ﻿hydroxyglutarate (2HG), a structural analog and competitive inhibitor of αKG. The production of 2HG is a common neomorphological activity of all IDH1 and 2 mutations, resulting in the block of αKG-dependent enzymes such as TET1 and 2 or histone demethylases causing aberrant DNA and histone methylation, altered gene expression profiles and successively impaired stem cell differentiation [9, 16, 17]. Consistent with a common pathogenic background, IDH1 and IDH2 mutation-positive AML cases share several clinical features, including older age at onset, higher platelet counts, and an association with intermediate cytogenetic risk [11, 14, 18–28].

Recently, several reviews have reported a poor prognosis for IDH1 mutations and a favorable prognosis for IDH2 mutations [29, 30]. However, the prognostic impact of IDH2R140 and IDH2R172 mutations in AML patients remains controversial and needs further evaluation. For example, IDH2R140 mutation has been shown to benefit the prognosis of AML patients in some studies [14, 31], whereas IDH2R140 mutation can shorten OS in AML patients in other studies [32, 33]. In addition, some studies cannot clarify the prognostic value of IDH2R140 mutation in AML [13, 34–39]. Similarly, IDH2R172 mutation favors the prognosis of AML patients in some studies [32, 33, 37]; however, in other studies, IDH2R172 mutation is detrimental to the prognosis of AML patients [38]. At the same time, some studies cannot clarify the prognostic value of IDH2R172 mutation in AML [13, 14, 31, 35, 36, 39]. Recently, IDH inhibitors have been identified as targeted therapies, and ivosidenib (IDH1 inhibitor) [40] and enasidenib (IDH2 inhibitor) [41] have shown promising results in patients with relapsed or refractory AML. They are being further investigated as monotherapies and in combination with multiple other established AML therapies. However, because the prognosis of IDH2 gene mutation subtypes in AML is controversial, it is imperative to clearly understand the prognostic value of each of these mutations, which will facilitate more accurate and personalized treatment. Therefore, we performed a meta-analysis on data from relevant published studies to further explore the comprehensive prognostic value of IDH2R140 and IDH2R172 mutations in AML patients.

## Methods

### Literature search

Two independent authors performed a comprehensive literature search of PubMed, Embase, and Cochrane as well as Chinese databases, including WanFang Database and China National Knowledge Internet (CNKI) databases, up to June 1, 2022. The terms included "AML", "acute myeloid leukemia", "IDH", "IDH2", "isocitrate dehydrogenase", and "isocitrate dehydrogenase 2". In addition, manual searches of the reference list were also performed. The review protocol has been registered in the PROSPERO International Prospective Register of Systematic Reviews (registration number: CRD42022344529).

### Selection of studies

Studies were included in the meta-analysis if they met the following criteria: (1) the study focused on the prognostic effect of IDH2R140 or IDH2R172 mutation in AML patients; (2) the study provided sufficient survival data, at least on overall survival (OS); (3) the hazard ratio (HR) and its 95% confidence interval (95% CI) were directly reported or could be calculated from original data; (4) the study included human subjects; and (5) the article was not a review, case report, or animal study. If the same or overlapping data were presented in multiple studies, only the most recent or the highest-quality study was included. The literature search and screening were conducted independently by 2 investigators. In case of disagreement, the opinions of the third investigator sought, and the best plan was determined after discussion.

### Data extraction

Two authors independently extracted the relevant data from the included articles. The following data were extracted from the articles: the first authors’ name, year of publication, study region, number of patients, median age and sex distribution of patients, incidence of different IDH gene mutations, mutation detection methods, cytogenetics, gene mutations, therapeutic regimens, data types and NOS scores. We selected OS as the primary endpoint and progression-free survival (PFS) as the secondary endpoint. OS was defined as the time between the first diagnosis and death or last follow-up for patients who were alive. PFS was defined as the time interval between the first diagnosis and relapse, progression, death, or last follow-up for patients alive in complete remission. If the article reports multiple HRs of univariate analysis and multivariate analysis, the result of multivariate analysis is given priority because they may be more accurate. When HR was not reported, we tried to contact the author to obtain it or used the method reported before to calculate it (extracting HR from the survival curve) [42].

### Quality assessment

The methodological quality of each included study was independently evaluated by two reviewers. We assessed the quality of cohort studies by applying the Newcastle‒Ottawa Scale (NOS) [43]. The NOS sums up to nine points, including selection (four points), comparability (two points), and exposure or outcome (three points). Studies that scored six or more points were regarded as high quality and eligible for our study. Divergences were resolved by discussion.

### Statistical analysis

All analyses were performed using STATA 15.0 (Stata Corporation, College Station, TX, USA). A bilateral *P* value of < 0.05 was defined as statistically significant. For OS and PFS, HRs and corresponding 95% CIs were used to assess the prognostic effect of IDH2R140 and IDH2R172 mutations in AML patients. In addition, compared to IDH2 wild-type AML patients, patients with IDH2 mutations had an HR > 1, suggesting a poorer prognosis. The Q test (*P* < 0.10 was considered significant heterogeneity) and I^2^ statistic (I^2^ = 0–25%: low heterogeneity; I^2^ = 25–50%: moderate heterogeneity; I^2^ = 50–100%: high heterogeneity) were used to test the heterogeneity of the included studies. When *P* > 0.1 or I^2^ < 50%, the heterogeneity of the study was considered to be not statistically significant, and a fixed-effects model was used for analysis; otherwise, the heterogeneity of the study was considered to be statistically significant, and the random effect model was used. We also analyzed the source of heterogeneity by subgroup analysis. Sensitivity analysis was used to investigate the influence of each study on the pooled HR, and Begg’s and Egger’s tests were conducted to detect the potential publication biases of the included studies.

## Results

### Study selection

**Fig. 1:**
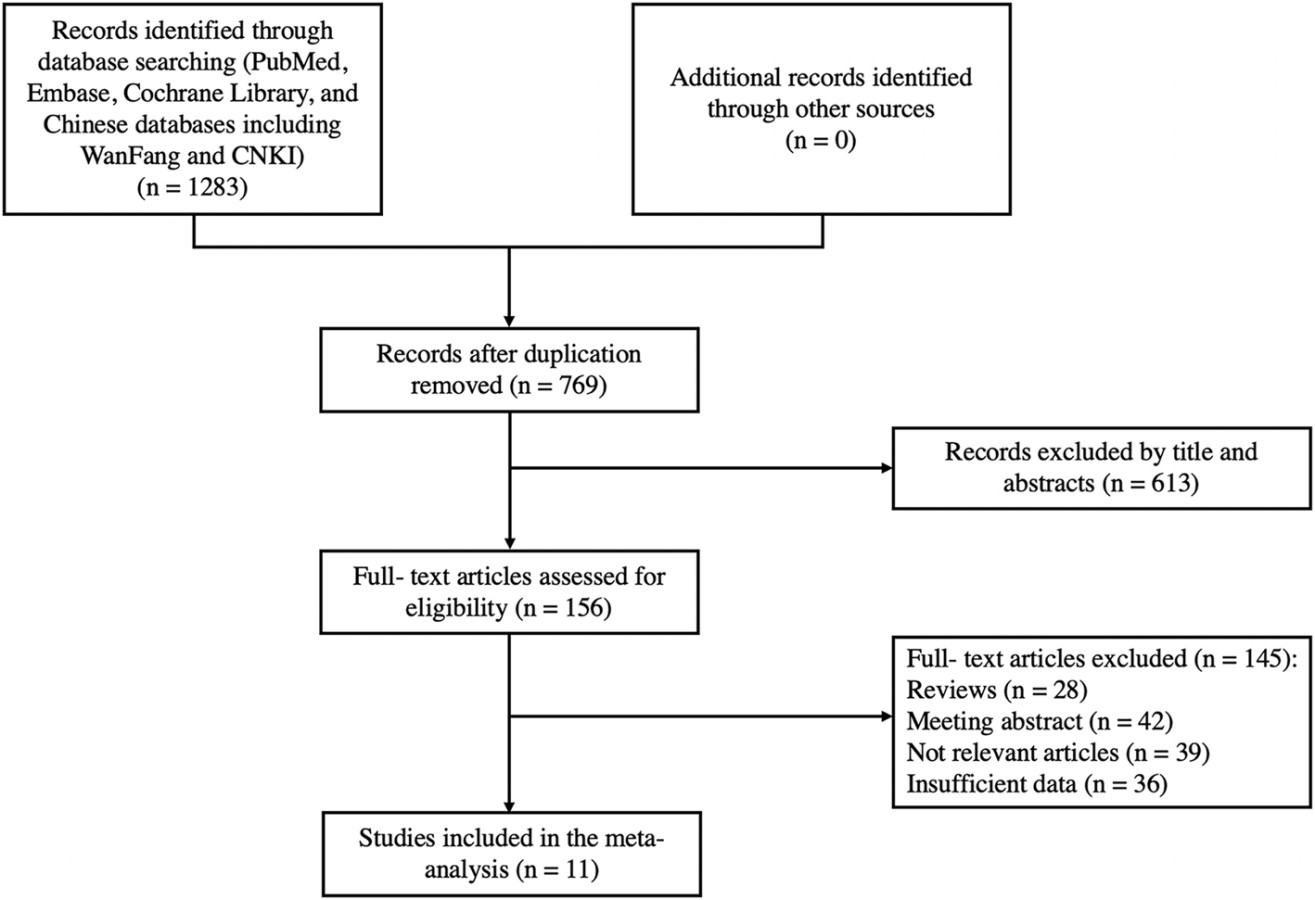
﻿Flow diagram of study selection

The literature screening process is shown in Fig. 1. Initially, 1283 articles were selected from four databases, and after removing duplicate articles, a total of 156 studies were obtained through a review of titles and abstracts based on inclusion and exclusion criteria. Next, 28 reviews and 42 meeting abstracts were removed; 39 studies were excluded because they were not relevant to the subject; and 36 studies were eliminated because of insufficient data. Finally, 11 studies that met the criteria were included in this meta-analysis [13, 14, 31–39] (Fig. 1).

### Characteristics of the included studies

Eleven studies including 12,725 AML patients were included in the meta-analysis, of which 1,416 (11.1%) patients had IDH2 mutations, while IDH2R140 and IDH2R172 mutations were found in 1,111 (8.7%) and 305 (2.4%) AML patients, respectively. The sample size ranged from 189 to 4930, and the frequencies of IDH2R140 and IDH2R172 mutations varied between 5.3 and 11.9% and between 0.7 and 5.1%, respectively. Four studies originated from Germany (one from Germany-Austria), two from the UK, two from the USA, one from China, one from Sweden, and one from Hungary. Seven studies had a median age greater than 50 years old, two studies were less than or equal to 50 years old, and the remaining two studies were unknown. Seven studies mainly used direct sequencing as the mutation detection method, and the other four studies used NGS. Patients in 6 eligible studies were classified by cytogenetics, while the other 5 studies were not. The cytogenetics of IDH2R140 were mainly favorable and intermediate risk, while IDH2R172 was predominantly intermediate risk. Nine studies analyzed combined mutations in patients with IDH2R140 and IDH2R172, while the remaining studies did not. The rate of combined NPM1 mutations was significantly higher in patients with IDH2R140 mutation than in those with IDH2R172 mutation. Moreover, most included studies showed that AML patients with IDH2R140 mutation had a higher FLT3-ITD mutation rate than those with IDH2R172 mutation. Furthermore, five studies showed the rate of allogeneic hematopoietic stem cell transplantation (allo-HSCT), while the other six studies did not. Among them, the allo-HSCT rates fluctuated between 12%-100% and 19.1%-100% in patients with IDH2R140 and IDH2R172 mutations, respectively. In addition, among the included studies, four and five studies obtained outcome indicators using multivariate analysis in IDH2R140 and IDH2R172, respectively. Notably, the results of the NOS scores of all included studies illustrated that all studies were of high quality (scores of 8–9). The characteristics are listed in Table 1.

**Table 1:** Characteristics of studies included in the meta-analysis

First Author	Year	Country	Sample size	Median age year (range)	Sex (male/female)	IDH1m	IDH2 m	IDH2 (R140) m	IDH2 (R172) m	Mutation detection methods	﻿Cytogenetics	Combined other gene mutations	Therapy-allo-HSCT	Data types	NOS
Bill [39]	2022	Germany	292	R140:66.5 (36.4–73.9) R172:64.5(47.1–74.8)	137/155	30 (10.3%)	41 (14.0%)	26 (8.9%)	15 (5.1%)	Direct sequencing and NGS	R140: fav 43%, int 35%, adv 22%; R172: fav 0%, int 67%, adv 33%;	R140: NPM1 43%, FLT3-ITD 30%, CEBPA 18%, TP53 0%; R172: NPM1 0%, FLT3-ITD 6.7%, CEBPA 14%, TP53 0%	R140:100% R172:100%	Others ^**a**^	9
Linch [38]	2021	UK	1204	R140:54(18–68) R172:57(31–70)	620/584	—	164 (13.6%)	120 (10.0%)	44 (3.6%)	Direct sequencing	Int 100%	R140: NPM1 53%, FLT3-ITD 27%; R172: NPM1 0%, FLT3-ITD 7%	R140:18% R172:34%	Others	9
Middeke [37]	2021	Germany	4930	R140:59(51–68) R172:61(50–66)	2501/2429	423 (8.6%)	575 (11.7%)	446 (9.0%)	110 (2.2%)	﻿Direct sequencing and NGS	R140: fav 43.7%, int 34.8%, adv 21.5%; R172: fav 2%, int 63%, adv 35%;	R140: NPM1 49.4%, FLT3-ITD 24.2%, CEBPA 3.8%; R172: NPM1 1.8%, FLT3-ITD 4.5%, CEBPA 5.5%	R140:14.6% R172:19.1%	Multivariate	9
Wu [36]	2021	China	389	< 60 years: IDH2 47.1%	218/171	24 (6.2)	34 (8.7%)	29 (7.5%)	5 (1.3%)	NGS	Unknown	Unknown	Unknown	Multivariate	8
Meggendorfer [33]	2018	Germany	1394	R140:66(21–90) R172:60(32–87)	730/664	110 (7.9%)	203 (14.6%)	166 (11.9%)	34 (2.4%)	NGS	Unknown	Unknown	R140:25% R172:54%	Others-R140 and multivariate-R172	9
Papaemmanuil [13]	2016	Germany-Austria	1540	— (18–84)	823/719	105 (6.8%)	146 (9.4%)	107 (6.9%)	39 (2.5%)	Exon sequencing	Unknown	NPM1 28%, CEBPA 5%, TP53 6%	Unknown	Multivariate	9
Willander [32]	2014	Sweden	189	R140:66(37–83) R172:72(46–74)	95/94	15 (7.9%)	26 (13.7%)	21 (11.1%)	5 (2,6%)	Direct sequencing	R140: fav 14.3%, int 57.1%, adv 19.0%; R172: fav 20%, int 20%, adv 60%;	R140: NPM1 38.1%, FLT3-ITD 19.0%; R172: NPM1 0%, FLT3-ITD 20%	Unknown	Multivariate	9
Koszarska [35]	2013	Hungary	376	R140:56.5(40–77) R172:56.5(31–66)	180/196	32 (8.5%)	28 (7.4%)	20 (5.3%)	8 (2.1%)	Direct sequencing	R140: fav 10.5%, int 79.0%, adv 10.5%; R172: fav 0%, int 87.5%, adv 12.5%;	R140: NPM1 47.4%, FLT3-ITD 20.0%; R172: NPM1 0%, FLT3-ITD 0%	Unknown	Others	9
JP. Patel [31]	2012	USA	657	48 (17–60)	335/322	46 (7.0%)	53 (8.1%)	39 (6.0%)	14 (2.1%)	Direct sequencing	Unknown	NPM1 22.3%, CEBPA 31.7%, TP53 12.4%	Unknown	Others	9
Green [14]	2011	UK	1473	R140:46(15–60) R172:47(29–62)	751/722	—	148 (10.1%)	119 (8.1%)	29 (2.0%)	Direct sequencing	R140: fav 5.0%, int 73.1%, adv 2.5%; R172: fav 0%, int 82.8%, adv 3.4%;	R140: NPM1 74.8%, FLT3-ITD 24.4%, CEBPA 5.0%; R172: NPM1 3.4%, FLT3-ITD 6.9%, CEBPA 6.7%	R140:12% R172:20%	Others	9
Ley [34]	2010	USA	281	53.1 (39.4–66.8)	152/129	25 (8.9%)	20 (7.1%)	18 (6.4%)	2 (0.7%)	WES, exome capture and sequencing	Unknown	R140: NPM1 27.8%, FLT3-ITD 5.6%; R172: NPM1 0%, FLT3-ITD 0%	Unknown	Others	9

*m* mutation, *NGS* next-generation sequencing, *allo-HSCT* allogeneic-hematopoietic stem cell transplantation, *fav* favorable, *int* intermediate, *adv* adverse, *WGS* whole-genome sequencing

^a^ Data extracted from calculated from numeric reports, univariate analyses, or Kaplan–Meier survival curves

### Prognostic impact of IDH2R140 mutation in patients with AML

**Fig. 2:**
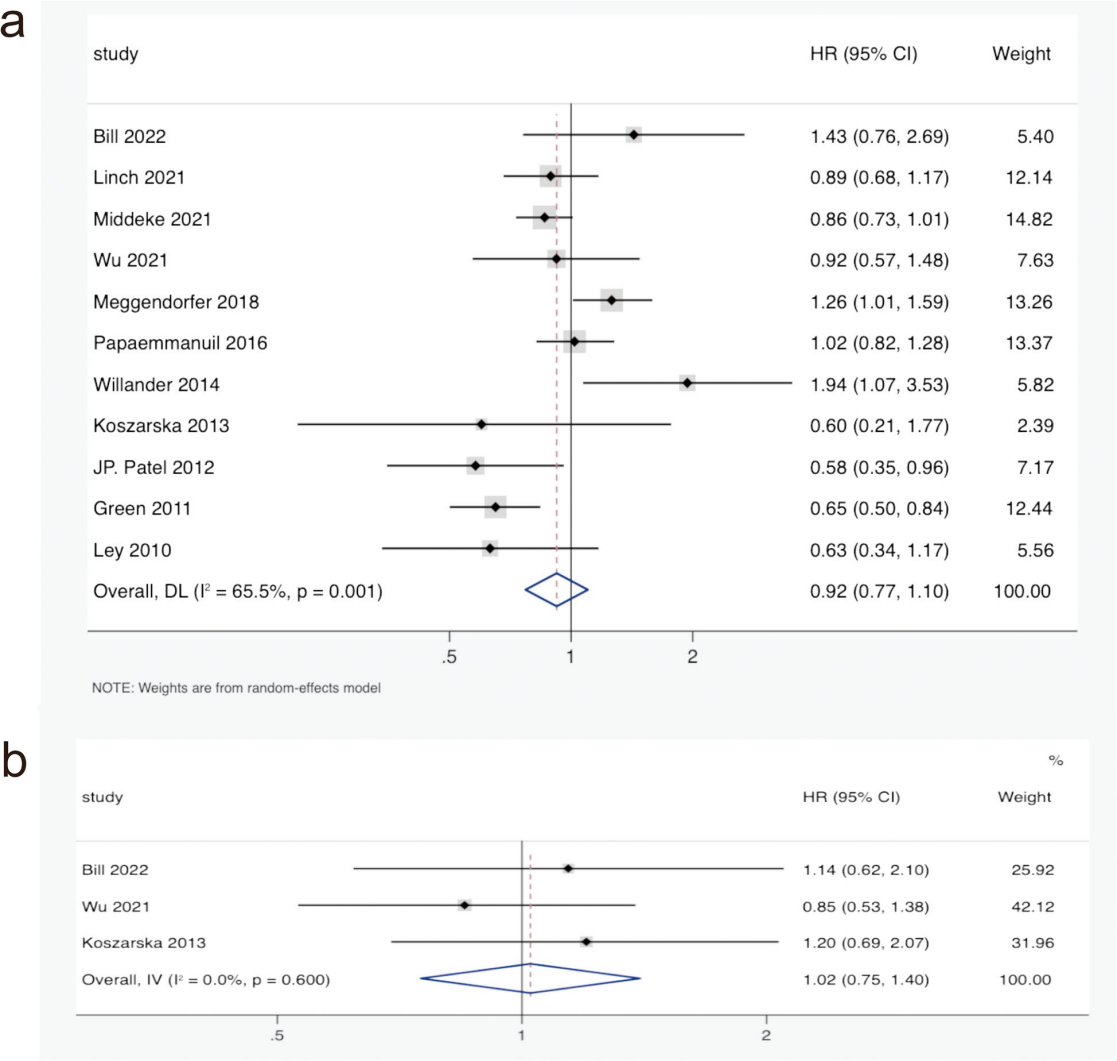
﻿Forest plot of the pooled HRs and 95% CIs assessing the prognostic value of IDH2R140 mutation in patients with AML. **a** For OS by a random-effects model. **b** For PFS by a fixed-effects model

There were 11 studies involving OS of IDH2R140 mutation in AML patients, including 12725 patients. As shown in Fig. 2a, the pooled HR for OS was 0.92 (95% CI: 0.77–1.10, *I*^*2*^ = 65.5%, *P* = 0.365) in AML patients with IDH2R140 mutation compared to those with IDH2R140 wild-type. The results indicated that the prognostic significance of IDH2R140 mutation in AML patients was unclear. The HRs of PFS were extracted from 3 eligible studies of IDH2R140 mutation in AML patients. The combined HR for PFS was 1.02 (95% CI: 0.75–1.40, *I*^*2*^ = 0.0%, *P* = 0.881) in IDH2R140-mutated AML patients compared with IDH2R140 wild-type patients (Fig. 2b). This finding also suggested that the effect of the IDH2R140 mutation on PFS in AML patients was unclear.

### Prognostic impact of IDH2R172 mutation in patients with AML

**Fig. 3:**
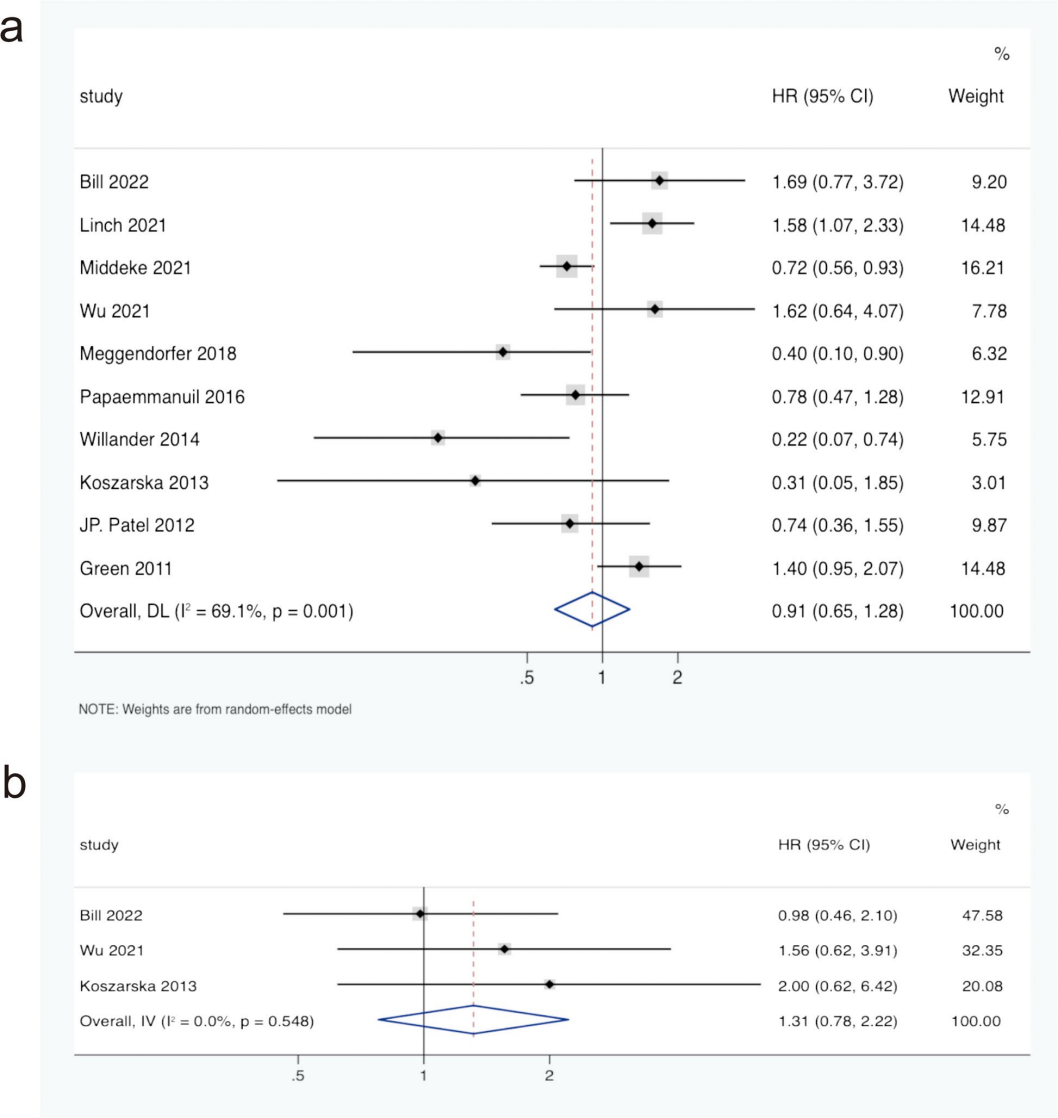
Forest plot of the pooled HRs and 95% CIs assessing the prognostic value of IDH2R172 mutation in patients with AML.** a** For OS by a random-effects model. **b** For PFS by a fixed-effects model

There were 10 studies involving OS of IDH2R172 mutation in AML patients, including 12444 patients. As shown in Fig. 3a, the pooled HR for OS was 0.91 (95% CI: 0.65–1.28, *I*^*2*^ = 69.1%, *P* = 0.590) in AML patients with IDH2R172 mutation compared to those with IDH2R172 wild-type. The prognostic significance of IDH2R172 mutation in AML patients was unclear. The HRs of PFS were also extracted from 3 eligible studies of IDH2R172 mutation in AML patients. The combined HR of PFS was 1.31 (95% CI: 0.78–2.22, *I*^*2*^ = 0.0%, *P* = 0.306) in IDH2R172-mutated AML patients compared with IDH2R172 wild-type patients (Fig. 3b). It also indicated that the effect of the IDH2R172 mutation on PFS in AML patients was unclear.

### Prognostic impact of IDH2R140 mutation vs IDH2R172 mutation in patients with AML

**Fig. 4:**
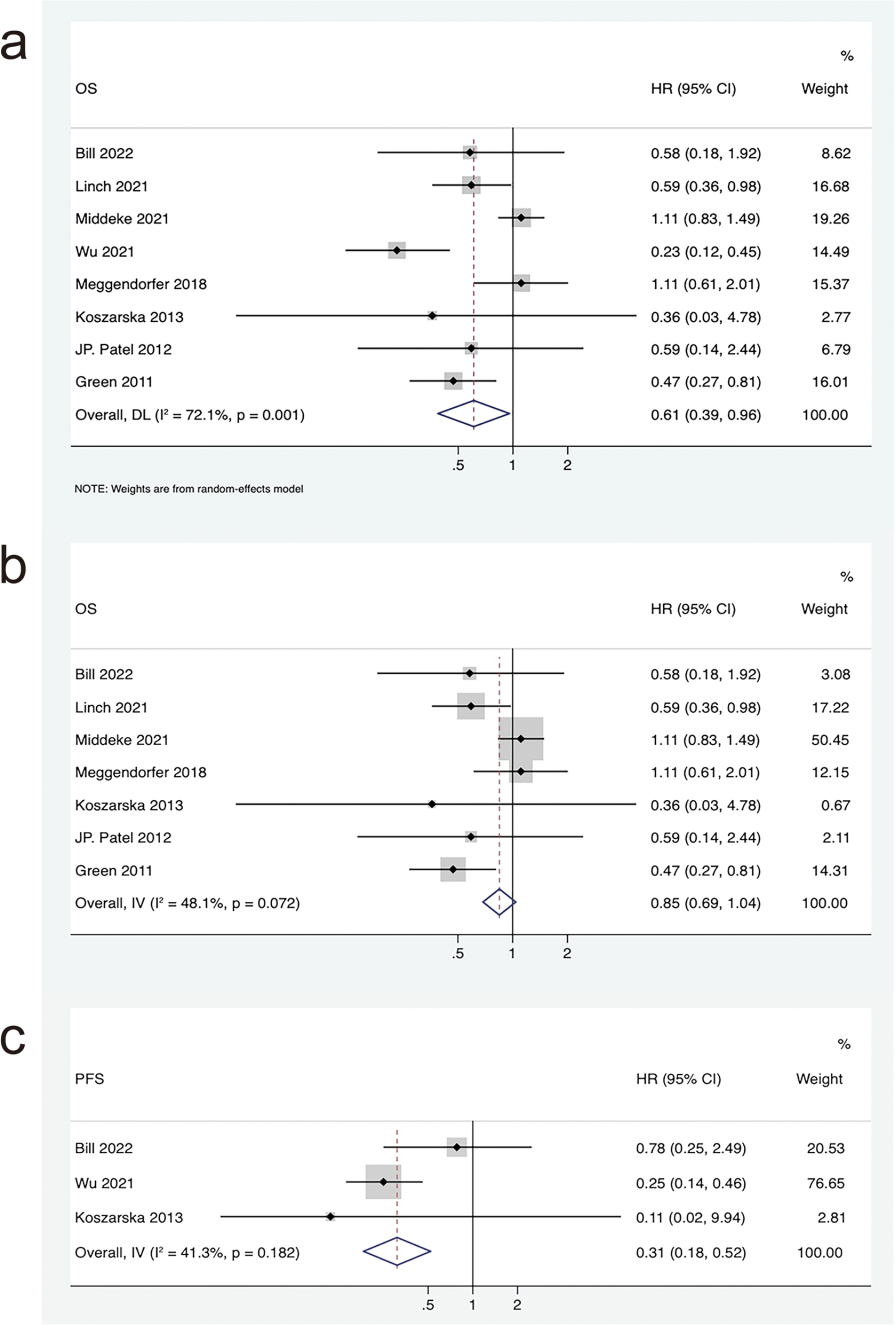
Forest plot of the pooled HRs and 95% CIs assessing the prognostic value of IDH2R140 mutation vs IDH2R172 mutation in patients with AML. **a** For OS by a random-effects model.** b** For OS by a fixed-effects model, after removing the study of Wu et al. 2021. **c** For PFS by a fixed-effects model

There were 8 studies involving OS of IDH2R140 mutation vs IDH2R172 mutation in AML patients, including 10,715 patients. As shown in Fig. 4a, the pooled HR of OS was 0.61 (95% CI: 0.39–0.96, I^2^ = 72.1%, *P* = 0.032) in AML patients with IDH2R140 mutation compared to those with IDH2R172 mutation. This suggested that patients with IDH2R140-mutated AML had a longer OS than those with IDH2R172-mutated AML, albeit with a high degree of heterogeneity. After removing the study of Wu et al. 2021 [36] with marked heterogeneity, the pooled HR of OS was 0.85 (95% CI: 0.69–1.04, I^2^ = 48.1%, *P* = 0.112) in AML patients with IDH2R140 mutation compared to those with IDH2R172 mutation (Fig. 4b). This showed that after removing studies with significant heterogeneity, although patients with AML with IDH2R140 mutation had longer OS than those with IDH2R172 mutation, there was no statistically significant difference. Additionally, HRs of PFS were also extracted from 3 eligible studies in IDH2R140 mutation vs IDH2R172 mutation of AML patients. The combined HR of PFS was 0.31 (95% CI: 0.18–0.52, I^2^ = 41.3%, *P* = 0.021) in IDH2R140-mutated AML patients compared with IDH2R172-mutated patients (Fig. 4c). This also indicated that IDH2R142-mutated AML patients had a longer PFS than IDH2R172-mutated AML patients, accompanied by a moderate degree of heterogeneity.

### Subgroup analysis and heterogeneity exploration

The results of the meta-analysis of the effect of IDH2R140 and IDH2R172 mutations on OS in AML patients showed that 11 studies and 10 studies had significant heterogeneity (*I*^*2*^ = 65.5% and 69.1%, respectively). We conducted subgroup analysis in terms of region, median age, mutation detection methods, cytogenetics, combined other gene mutations, the rate of allo-HSCT, and data types to check the heterogeneity and to determine whether the above factors will change the relationship between IDH2R140 and IDH2R172 mutations and OS (Table 2).

**Table 2:** Subgroup analyses of OS on IDH2R140 mutation and the IDH2R172 mutation

Comparison variables	IDH2R140 mutation				IDH2R172 mutation			
	No. of studies	Pooled HR (95%CI)	*P*	I^2^(%), *P*_h_	No. of studies	Pooled HR (95%CI)	*P*	I^2^(%), *P*_h_
Total	11	0.92 (0.77–1.10)	0.365	65.6, 0.001	10	0.91 (0.65–1.28)	0.590	69.1, 0.001
Region								
Germany /Austria	4	1.05 (0.85–1.30)	0.630	65.0, 0.036	4	0.76 (0.61–0.94)	0.012	45.0, 0.142
UK	2	0.76 (0.56–1.03)	0.079	62.9, 0.101	2	1.49 (1.13–1.96)	0.005	0.0, 0.667
China	1	0.92 (0.57–1.48)	0.719	/	1	1.62 (0.64–4.07)	0.306	/
Swedish	1	1.94 (1.07–3.53)	0.030	/	1	0.22 (0.07.74)	0.014	/
Hungary	1	0.60 (0.21–1.77)	0.357	/	1	0.31 (0.05–1.85)	0.179	/
USA	2	0.60 (0.41–0.89)	0.010	0.0, 0.839	1	0.74 (0.36–1.55)	0.305	/
Median age								
≤ 50	2	0.63 (0.50–0.80)	0.000	0.0, 0.694	2	1.10 (0.60–2.02)	0.757	56.2, 0.131
> 50	7	1.02 (0.81-.123)	0.839	64.6, 0.010	6	0.76 (0.43–1.34)	0.338	77.6, 0.000
Unknown	2	1.00 (0.82–1.23)	0.990	0.0, 0.701	2	0.92 (0.59–1.43)	0.713	46.1, 0.173
Mutation detection methods								
Direct sequencing	7	0.87 (0.69–1.11)	0.260	64.2, 0.010	7	0.93 (0.60–1.43)	0.745	75.8, 0.000
NGS	4	1.07 (0.93–1.24)	0.342	44.2, 0.146	3	0.82 (0.54–1.23)	0.342	68.7, 0.012
Therapy								
Allo-HSCT	5	0.93 (0.73–1.19)	0.581	76.2, 0.002	5	1.09 (0.69–1.72)	0.781	78.9, 0.001
Unknown	6	0.90 (0.65–1.23)	0.502	58.3, 0.035	5	0.82 (0.44–1.50)	0.514	68.7, 0.012
Data type								
Multivariate	4	1.01 (0.80–1.28)	0.915	59.1, 0.062	5	0.69 (0.46–1.04)	0.078	50.2, 0.090
Others ^a^	7	0.84 (0.63–1.12)	0.233	71.8, 0.002	5	1.35 (1.06–1.73)	0.014	34.7, 0.190

*P*_h_ *P* value for heterogeneity, F Fixed-effects model, R Random-effects model, NGS next-generation sequencing

^a^ Data extracted from calculated from numeric reports, univariate analyses, or Kaplan–Meier survival curves

According to the region of the studies, we found a significant association between IDH2R140 mutation and OS in the USA (HR = 0.60, 95% CI: 0.41–0.89, *I*^*2*^ = 0.0%, *P* = 0.010) and Sweden (HR = 1.94, 95% CI: 1.07–3.53, *P* = 0.030) but not in Germany/Austria (HR = 1.05, 95% CI: 0.85–1.30, *I*^*2*^ = 65.5%, *P* = 0.630), the UK (HR = 0.76, 95% CI: 0.56–1.03, *I*^*2*^ = 62.9%, P = 0.079), China (HR = 0.92, 95% CI: 0.57–1.48, *P* = 0.719), or Hungary (HR = 0.60, 95% CI: 0.21–1.77, *P* = 0.357). We observed a significantly longer OS in AML patients with IDH2R140 mutation than in those with IDH2R140 wild-type in the subgroup of median age ≤ 50 years old (HR = 0.63, 95% CI: 0.50–0.80, *I*^*2*^ = 0.0%, *P* = 0.000). Other subgroup analyses did not find a significant association of IDH2R140 mutation with OS in AML patients.

Regarding the association between IDH2R172 mutation and OS, according to the region of the included studies, we found a significant association in Germany/Austria (HR = 0.76, 95% CI: 0.61–0.94, *I*^*2*^ = 45.0%, *P* = 0.012), the UK (HR = 1.49, 95% CI: 1.13–1.96, *P* = 0.005), and Sweden (HR = 0.22, 95% CI: 0.07–0.74, *I*^*2*^ = 62.9%, *P* = 0.014) but not in China (HR = 1.62, 95% CI: 0.64–4.07, *P* = 0.306), Hungary (HR = 0.31, 95% CI: 0.05–1.85, *P* = 0.179), or the USA (HR = 0.74, 95% CI: 0.36–1.55, *P* = 0.305). Additionally, we observed a significantly shorter OS in AML patients with IDH2R172 mutation than in those with IDH2R172 wild-type in the subgroup of other data types (HR = 1.35, 95% CI: 1.06–1.73, *I*^*2*^ = 34.7%, *P* = 0.014). Surprisingly, in the multivariate analysis subgroup, AML patients with IDH2R172 mutation had a longer OS than patients with IDH2R172 wild-type (HR = 0.69, 95% CI: 0.46–1.72, *I*^*2*^ = 50.2%, *P* = 0.078), although the difference was not statistically significant. Likewise, other subgroup analyses did not find a significant association of IDH2R172 mutation with OS in AML patients.

### Sensitivity analysis and publication bias

**Fig. 5:**
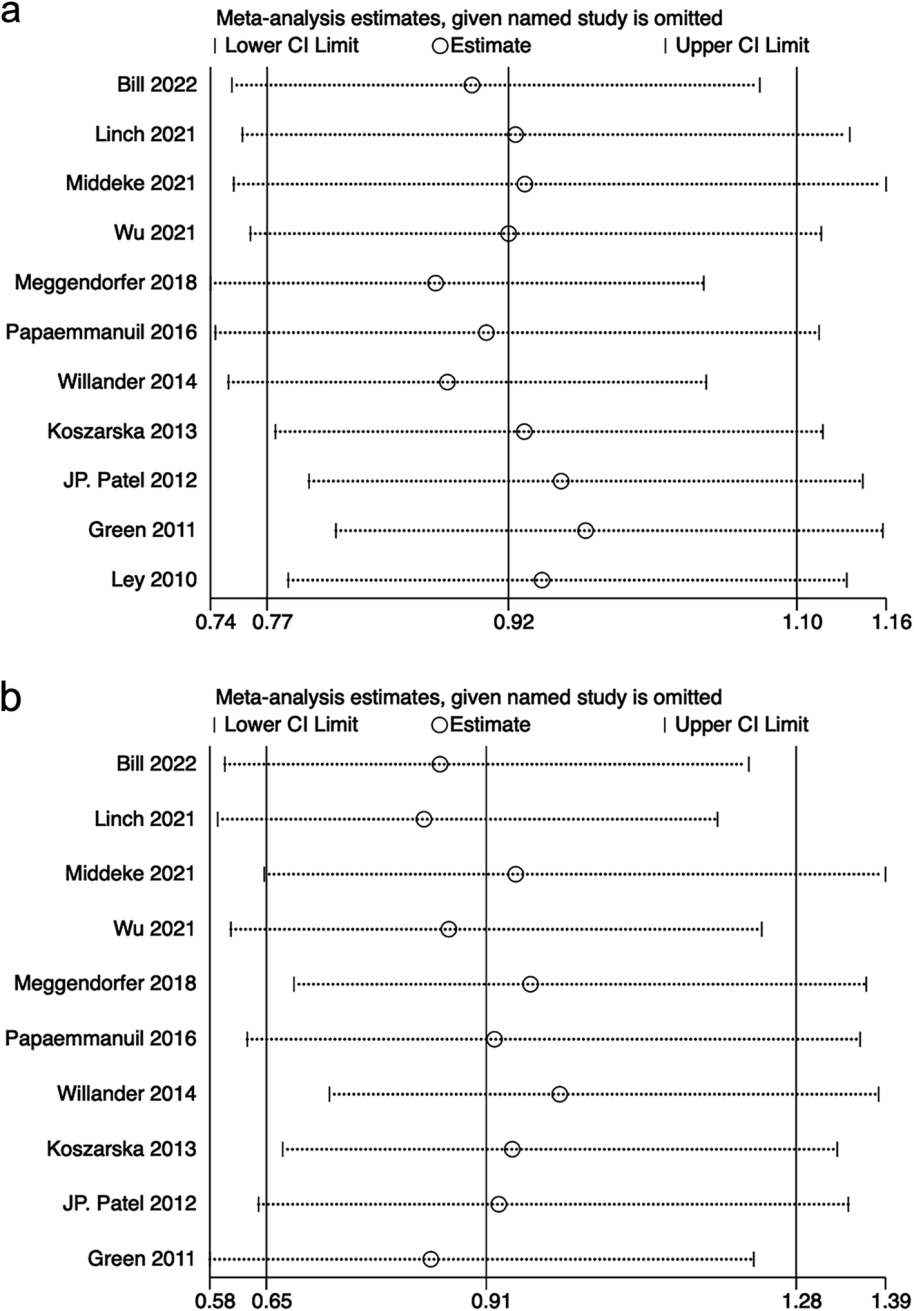
Sensitivity analysis for OS in patients with AML. **a** IDH2R140 mutation. **b** IDH2R172 mutation

We conducted sensitivity analyses of 11 and 10 studies describing the relationship between IDH2R140 and IDH2R172 mutations and OS to validate the stability of the meta-analysis, respectively. As shown in Fig. 5a and b, sensitivity analyses showed that no individual study had a predominant effect on the pooled HR, indicating that the results were stable and reliable. In addition, Begg’s and Egger’s tests were used to detect publication biases, which indicated that there was no significant bias between studies of IDH2R140 mutation (*P* = 1.000 of Begg’s test and *P* = 0.991 of Egger’s test) and of IDH2R172 mutation (*P* = 0.283 of Begg’s test and *P* = 0.625 of Egger’s test).

## Discussion

With further development of sequencing technology, prognostic stratification of AML disease with high heterogeneity has become more precise. Here, we performed a meta-analysis of the prognostic impact of IDH2 gene mutation subtypes R140 and R172 on AML patients. This meta-analysis included 12725 AML patients from eleven different centers, of whom 1416 (11.1%) had IDH2 mutations, while 1111 (8.7%) and 305 (2.4%) had IDH2R140 and IDH2R172 mutations, respectively. Our results showed that, except for certain subgroup analyses, IDH2R140 and IDH2R172 mutations had no significant effect on OS and PFS in AML patients. AML patients with IDH2R140 mutation have a better prognosis than those with IDH2R172 mutation, albeit with some degree of heterogeneity. The study is the most recent and has the largest sample size in the included literature to date on the impact of IDH2 gene mutation subtypes on the prognosis of AML patients. The study truly reflects the real-world situation, and the findings are highly informative.

Interestingly, we found that geography and age significantly influenced the prognosis of AML patients in the IDH2R140 gene mutation subgroup analysis. That is, IDH2R140 mutation improved OS in USA AML patients (USA, HR: 0.60, *P* = 0.010) and younger patients (median age ≤ 50 years, HR: 0.63, *P* = 0.000). However, it shortened OS in AML patients from Sweden (Sweden, HR: 1.94, *P* = 0.030). Similarly, we found that region and data type significantly influenced the prognosis of AML patients in the IDH2R172 gene mutation subgroup analysis. Namely, IDH2R172 mutation improved OS in AML patients from Germany/Austria (Germany/Austria, HR: 0.76, *P* = 0.012) and Sweden (Sweden, HR: 0.22, *P* = 0.014). However, it shortened OS in AML patients from the UK and AML patients with nonmultifactorial analysis data (others, HR: 1.35, *P* = 0.014). Therefore, our findings are partially similar to the results of the meta-analysis of Xu et al. 2017 [30], which showed that IDH2R140 mutation improves OS in younger AML patients, with significant heterogeneity in the prognostic value of IDH2R172. At the same time, we have some new findings that among patients with IDH2R140 and IDH2R172 mutations, regional differences also have a significant impact on prognosis. Moreover, differences in data types also have a significant impact on the prognosis of patients with IDH2R172 mutation. These results are the results we found first.

Additionally, our study found that IDH2R140-mutated AML patients had significantly longer OS (HR = 0.61, 95% CI: 0.39–0.96, I^2^ = 72.1%, *P* = 0.032) and PFS (HR = 0.31, 95% CI: 0.18–0.52, I^2^ = 41.3%, *P* = 0.021) than IDH2R172-mutated patients, albeit with some degree of heterogeneity. Among them, the study of Wu et al. 2021 is the largest study on OS heterogeneity. After removing the study of Wu et al. 2021, the pooled HR heterogeneity was less than 50%, and the OS (HR = 0.85, 95% CI: 0.69–1.04, I^2^ = 48.1%, *P* = 0.112) of AML patients with IDH2R140 mutation was still longer than that of IDH2R172 mutation patients, but the difference was not statistically significant. The study by Wu et al. 2021 from China is characterized by a small number of cases (29 patients with IDH2R140 mutation and 5 patients with IDH2R172 mutation), and the median age, cytogenetic classification, proportion of co-mutated genes, and specific treatment are unknown. This was significantly different from the other 7 studies. These differences may be the source of its heterogeneity.

Furthermore, in terms of clinical characteristics, our study also found an IDH2 mutation rate of 11.1%, similar to previous findings [12–14]. The cytogenetics of AML patients with IDH2R140 mutation was mainly favorable and intermediate risk, while IDH2R172 mutation was mainly intermediate risk. Moreover, IDH2R140 mutation was more likely to coexist with NPM1 mutation, and IDH2R172 mutation was mutually exclusive with NPM1 and rarely accompanied by other gene mutations. These findings are also consistent with previous studies [14, 30, 31, 33, 35–39]. Disappointingly, in the included studies, no subgroup analysis was performed on the prognostic effects of cytogenetic classification, different treatment regimens, and co-mutation gene status in patients with IDH2R140- and IDH2R172-mutated AML, so we could not summarize them.

In our study, the heterogeneity of the OS meta-analysis was high in AML patients with IDH2R140 and IDH2R172 mutations. Heterogeneity was examined by removing one study at a time, and no studies were found that had a significant effect on prognosis in the meta-analysis. However, by performing subgroup analysis on the variables, we found that heterogeneity could be significantly reduced by performing subgroup analysis on region, age, and data type. Therefore, the heterogeneity of our study can be explained to some extent by geography, age, and data type.

Despite our efforts to refine this meta-analysis, it still has its own limitations. First, although the included studies were searched from mainstream English and Chinese databases, other relevant studies, especially those published in non-English or non-Chinese languages or not publicly available, might be overlooked. Publication bias could not be completely avoided. Second, our analysis was based on observational studies rather than prospective controlled studies or randomized trials, and the selection criteria were difficult to grasp; the homogeneity of the studies was difficult to ensure. Therefore, the evidence strength of evidence-based medicine in this paper is not high. Third, some of the data are calculated from univariate analysis or Kaplan‒Meier survival curves or numerical reports, which is crude, and may differ slightly from the facts. Fourth, the heterogeneity of OS was high in both whole group and some subgroups, which may be related to the different clinical characteristics of each study. Finally, although we extracted as many HRs as possible from multivariate analyses, there were various confounding factors (different cytogenetic classes, differences in treatment regimens, and differences in co-mutated gene status), which may also be the source of heterogeneity.

## Conclusion

In conclusion, this meta-analysis reveals that IDH2R140 mutation improves OS in younger AML patients and that the prognostic value of IDH2R172 mutations is significantly heterogeneous. Differences in region and data type have a significant impact on the prognosis of AML patients with IDH2R140 and/or IDH2R172 mutations. In addition, AML patients with IDH2R140 mutation have a better prognosis than those with IDH2R172 mutation, albeit with some degree of heterogeneity. However, due to the limitations of the original study, our conclusions await further validation in a larger sample size, multicenter, randomized controlled design prospective study.

## Data Availability

All data generated or analysed during this study are included in this published article.

## Abbreviations

IDH2: Isocitrate dehydrogenase 2
IDH1: Isocitrate dehydrogenase 1
AML: Acute myeloid leukemia
HR: Hazard ratio
CI: Confidence intervals
PFS: Progression-free survival
IR-AML: AML patients with intermediate risk karyotypes
ITD: Internal tandem duplication
FLT3: Fms-like tyrosine kinase 3
NPM1: Nucleophosmin 1
NSG: Next-generation sequencing
DNMT3A: DNA methyltransferase 3A
TET2: Tet oncogene family member 2
αKG: α-Ketoglutarate
2HG: 2 Hydroxyglutarate
CNKI: China national knowledge internet
NOS: Newcastle‒Ottawa scale
allo-HSCT: Allogeneic hematopoietic stem cell transplantation

## References

[1] Short NJ, Konopleva M, Kadia TM (2020). Advances in the treatment of acute myeloid leukemia: new drugs and new challenges. Cancer Discov.

[2] Dohner H, Estey E, Grimwade D (2017). Diagnosis and management of AML in adults: 2017 ELN recommendations from an international expert panel. Blood.

[3] Grimwade D, Hills RK, Moorman AV (2010). Refinement of cytogenetic classification in acute myeloid leukemia: determination of prognostic significance of rare recurring chromosomal abnormalities among 5876 younger adult patients treated in the United Kingdom Medical Research Council trials. Blood.

[4] Falini B, Mecucci C, Tiacci E (2005). Cytoplasmic nucleophosmin in acute myelogenous leukemia with a normal karyotype. New England J Med.

[5] Bienz M, Ludwig M, Mueller BU (2005). Risk assessment in patients with acute myeloid leukemia and a normal karyotype. Clin Cancer Res.

[6] Nakao M, Yokota S, Iwai T (1996). Internal tandem duplication of the flt3 gene found in acute myeloid leukemia. Leukemia.

[7] Mrozek K, Marcucci G, Paschka P, Whitman SP, Bloomfield CD (2007). Clinical relevance of mutations and gene-expression changes in adult acute myeloid leukemia with normal cytogenetics: are we ready for a prognostically prioritized molecular classification?. Blood.

[8] Ilyas AM, Ahmad S, Faheem M (2015). Next Generation sequencing of acute myeloid leukemia: influencing prognosis. Bmc Genomics.

[9] Figueroa ME, Abdel-Wahab O, Lu C (2010). Leukemic IDH1 and IDH2 mutations result in a hypermethylation phenotype, disrupt TET2 function, and impair hematopoietic differentiation. Cancer Cell.

[10] Xu J, Wang YY, Dai YJ (2014). DNMT3A Arg882 mutation drives chronic myelomonocytic leukemia through disturbing gene expression/DNA methylation in hematopoietic cells. Proceed Natl Acad Sci United States America.

[11] Mardis ER, Ding L, Dooling DJ (2009). Recurring Mutations found by sequencing an acute myeloid leukemia genome. New England J Med.

[12] Ley TJ, Miller C, Ding L (2013). Genomic and Epigenomic landscapes of adult de novo acute myeloid leukemia. New England J Med.

[13] Papaemmanuil E, Gerstung M, Bullinger L (2016). Genomic classification and prognosis in acute myeloid leukemia. New England J Med.

[14] Green CL, Evans CM, Zhao L (2011). The prognostic significance of IDH2 mutations in AML depends on the location of the mutation. Blood.

[15] Dang LN, Jin SF, Su SSM (2010). IDH mutations in glioma and acute myeloid leukemia. Trends Mol Med.

[16] Lu C, Ward PS, Kapoor GS (2012). IDH mutation impairs histone demethylation and results in a block to cell differentiation. Nature.

[17] Xu W, Yang H, Liu Y (2011). Oncometabolite 2-Hydroxyglutarate is a competitive inhibitor of alpha-ketoglutarate-dependent dioxygenases. Cancer Cell.

[18] Chotirat S, Thongnoppakhun W, Promsuwicha O, Boonthimat C, Auewarakul CU (2012). Molecular alterations of isocitrate dehydrogenase 1 and 2 (IDH1 and IDH2) metabolic genes and additional genetic mutations in newly diagnosed acute myeloid leukemia patients. J Hematol Oncol.

[19] Chou WC, Lei WC, Ko BS (2011). The prognostic impact and stability of Isocitrate dehydrogenase 2 mutation in adult patients with acute myeloid leukemia. Leukemia.

[20] Wagner K, Damm F, Gohring G (2010). Impact of IDH1 R132 mutations and an IDH1 single nucleotide polymorphism in cytogenetically normal acute myeloid leukemia: SNP rs11554137 is an adverse prognostic factor. J Clin Oncol.

[21] Thol F, Damm F, Wagner K (2010). Prognostic impact of IDH2 mutations in cytogenetically normal acute myeloid leukemia. Blood.

[22] Schnittger S, Haferlach C, Ulke M (2010). IDH1 mutations are detected in 6.6% of 1414 AML patients and are associated with intermediate risk karyotype and unfavorable prognosis in adults younger than 60 years and unmutated NPM1 status. Blood.

[23] Paschka P, Schlenk RF, Gaidzik VI (2010). IDH1 and IDH2 mutations are frequent genetic alterations in acute myeloid leukemia and confer adverse prognosis in cytogenetically normal acute myeloid leukemia with NPM1 mutation without FLT3 internal tandem duplication. J Clin Oncol.

[24] Marcucci G, Maharry K, Wu YZ (2010). IDH1 and IDH2 gene mutations identify novel molecular subsets within de novo cytogenetically normal acute myeloid leukemia: a cancer and leukemia group B study. J Clin Oncol.

[25] Green CL, Evans CM, Hills RK (2010). The prognostic significance of IDH1 mutations in younger adult patients with acute myeloid leukemia is dependent on FLT3/ITD status. Blood.

[26] Chou WC, Hou HA, Chen CY (2010). Distinct clinical and biologic characteristics in adult acute myeloid leukemia bearing the isocitrate dehydrogenase 1 mutation. Blood.

[27] Boissel N, Nibourel O, Renneville A (2010). Prognostic impact of isocitrate dehydrogenase enzyme isoforms 1 and 2 mutations in acute myeloid leukemia: a study by the acute leukemia french association group. J Clin Oncol.

[28] Abbas S, Lugthart S, Kavelaars FG (2010). Acquired mutations in the genes encoding IDH1 and IDH2 both are recurrent aberrations in acute myeloid leukemia: prevalence and prognostic value. Blood.

[29] Zhou KG, Jiang LJ, Shang Z (2012). Potential application of IDH1 and IDH2 mutations as prognostic indicators in non-promyelocytic acute myeloid leukemia: a meta-analysis. Leukemia Lymphoma.

[30] Xu QY, Li Y, Lv N (2017). Correlation between isocitrate dehydrogenase gene aberrations and prognosis of patients with acute myeloid leukemia: a systematic review and meta-analysis. Clin Cancer Res.

[31] Patel JP, Gonen M, Figueroa ME (2012). Prognostic relevance of integrated genetic profiling in acute myeloid leukemia. New England J Med.

[32] Willander K, Falk IJ, Chaireti R (2014). Mutations in the isocitrate dehydrogenase 2 gene and IDH1 SNP 105C>T have a prognostic value in acute myeloid leukemia. Bio Res.

[33] Meggendorfer M, Cappelli LV, Walter W (2018). DH1R132, DH2R140 and 1DH2R172 in AML: different genetic landscapes correlate with outcome and may influence targeted treatment strategies. Leukemia.

[34] Ley TJ, Ding L, Walter MJ (2010). DNMT3A Mutations in Acute Myeloid Leukemia. New England J Med.

[35] Koszarska M, Bors A, Feczko A (2013). Type and location of isocitrate dehydrogenase mutations influence clinical characteristics and disease outcome of acute myeloid leukemia. Leukemia Lymphoma..

[36] Wu RY, Xie XS, Wei Y (2021). Prognostic significance of different IDH mutations and accompanying gene mutations in patients with acute myeloid leukemia. Zhonghua xue ye xue za zhi = Zhonghua xueyexue zazhi.

[37] Middeke JM, Metzeler KH, Rollig C (2022). Differential impact of IDH1/2 mutational subclasses on outcome in adult AML: results from a large multicenter study. Blood Adv.

[38] Linch DC, Hills RK, Burnett AK, Russell N, Gale RE (2022). Therapy for isocitrate dehydrogenase 2 (IDH2)(R172)-mutant acute myeloid leukaemia. British J Haematol.

[39] Bill M, Jentzsch M, Bischof L (2022). Impact of IDH1 and IDH2 mutation detection at diagnosis and in remission in patients with AML receiving allogeneic transplantation. Blood Adv.

[40] DiNardo CD, Stein EM, de Botton S (2018). Durable remissions with Ivosidenib in IDH1-mutated relapsed or refractory AML. New England J Med.

[41] Stein EM, DiNardo CD, Pollyea DA (2017). Enasidenib in mutant IDH2 relapsed or refractory acute myeloid leukemia. Blood.

[42] Wang Y, Zeng TT (2013). Response to: practical methods for incorporating summary time-to-event data into meta-analysis. Trials.

[43] Stang A (2010). Critical evaluation of the Newcastle-Ottawa scale for the assessment of the quality of nonrandomized studies in meta-analyses. Eur J Epidemiol.

